# Human Cardioviruses, Meningitis, and Sudden Infant Death Syndrome in Children

**DOI:** 10.3201/eid1712.111037

**Published:** 2011-12

**Authors:** Jan Felix Drexler, Sigrid Baumgarte, Monika Eschbach-Bludau, Arne Simon, Christoph Kemen, Udo Bode, Anna-Maria Eis-Hübinger, Burkhard Madea, Christian Drosten

**Affiliations:** University of Bonn Medical Centre, Bonn, Germany (J.F. Drexler, M. Eschbach-Bludau, A. Simon, U. Bode, A.-M. Eis-Hübinger, B. Madea, C. Drosten);; Institute of Hygiene and the Environment, Hamburg, Germany (S. Baumgarte);; Catholic Children's Hospital Wilhelmstift, Hamburg (C. Kemen)

**Keywords:** Picornaviridae infections, human cardiovirus, Germany, communicable diseases, meningitis, children, real-time RT-PCR, sudden infant death syndrome, viruses

## Abstract

Cardioviruses cause myocarditis and encephalomyelitis in rodents; human cardioviruses have not been ascribed to any disease. We screened 6,854 cerebrospinal fluid and 10 myocardium specimens from children and adults. A genotype 2 cardiovirus was detected from a child who died of sudden infant death syndrome, and 2 untypeable cardioviruses were detected from 2 children with meningitis.

The cardioviruses (family *Picornaviridae*, genus *Cardiovirus*) are pathogens of rodents and include a murine encephalomyocarditis virus and Theiler’s virus and related strains (species *Theilovirus*), the latter serving as laboratory models of the pathogenesis of multiple sclerosis in mice (*1*). The existence of specific human cardioviruses was suspected in the 1960s in conjunction with a rare infectious neurodegenerative disease known as Vilyuisk encephalitis (*2**,**3*). Recently, human cardioviruses (hCVs) were identified in archived diagnostic cell culture supernatants (*4*) and in clinical samples from children with diarrhea or respiratory infection (*5**,**6*). Up to 8 different putative hCV types have since been characterized in human feces (*7*).

Despite the remarkable pathogenicity of rodent cardioviruses, specific disease associations of hCV could not be made. An initial clinical study yielded no evidence of hCV in cerebrospinal fluid (CSF) of 400 patients with aseptic meningitis, encephalitis, or multiple sclerosis (*8*). To evaluate the pathogenetic potential of these emerging viruses, we investigated 6,854 CSF specimens from adults and children with neurologic disease and 10 myocardium specimens from infants who had died of sudden infant death syndrome (SIDS).

## The Study

CSF specimens were collected from 3 cohorts. The first cohort comprised 2,562 specimens sent during 1998–2008 to the Institute of Virology, University of Bonn Medical Center (UBMC), Bonn, Germany, for routine investigation of meningoencephalitis (333 from the Department of Pediatrics and 2,229 from other departments). The second cohort comprised 3,960 specimens collected during 1982–2008 at the UBMC children’s hospital from children with cancer and neurologic complications during chemotherapy. The third cohort comprised 348 specimens from hospitalized children with clinical meningitis or encephalitis in which no etiologic agent had been found; the specimens were sent for virologic investigation to the Institute for Hygiene and the Environment in Hamburg, ≈400 km from UBMC, during 2006–2008. Myocardium specimens were collected during 2010 at the UBMC Institute for Forensic Medicine from 10 epidemiologically unlinked children who died of SIDS.

Viral RNA was purified from clinical specimens by using the Viral RNA Mini and RNeasy Mini kits (QIAGEN, Hilden, Germany). Detection of hCV RNA was done in pools of 2–10 specimens by using quantitative real-time reverse transcription PCR (RT-PCR) and nested RT-PCR specific for the viral 5′ untranslated region (5′-UTR), as described (*6*). Amplification of further hCV genomic regions from individual positive specimens was conducted by using ≈20 sets of different nested RT-PCRs (primers available on request from C.D.).

In 2 of 681 CSF specimens (n = 333 and n = 348 from cohorts 1 and 3, respectively) from children with meningitis (Table A1), hCV RNA was detected at low concentrations (1.14 × 10^4^ and 9.63 × 10^2^ copies/mL). In 1 of these patients, hCV was also detectable in feces (9.50 × 10^2^ copies/g). In 1 of 10 myocardium specimens, hCV was detected by nested RT-PCR, and results of quantitative real-time RT-PCR were negative. Underquantification because of nucleotide mismatches below oligonucleotide binding sites and contamination of nested RT-PCR was excluded by sequence comparison (up to 5% nt divergence from other hCV strains, including the positive control). Serum and liver specimens from the patient who died of SIDS were negative according to real-time RT-PCR. No histopathologic alterations could be observed in myocardial tissue from this same patient.

To evaluate whether detected hCV strains differed from previously described genotypes, amplification and nucleotide sequencing of additional genomic regions was attempted. In a case of meningoencephalitis (specimen 07/03981), we sequenced a 1,297-nt fragment comprising the near complete 5′-UTR and the first 489 nt of the structural protein gene (leader, viral protein [VP] 4 domain, and upstream VP2 domain, GenBank accession no. JN209931). Despite repeated trials, further sequence fragments could be amplified neither from the specimen from this patient nor from that from the second patient with meningoencephalitis that showed very low virus concentrations (specimen VI1607). From the specimen from the SIDS patient (specimen 347/10), amplification of the complete structural genome and partial nonstructural genome was successful (5,333 nt, GenBank accession no. JN209932). This virus belonged to hCV genotype 2 in the VP1 genomic region (i.e., the region used for the designation of genotypes) (Figure, panel A). The CSF specimen 07/03981 was also phylogenetically related to genotype 2 viruses in the 5′-UTR and Leader-VP2 genomic regions (Figure, panels B and C). On the basis of the 5′-UTR sequences, the closest known relative to both viruses was D/VI2229, obtained in Germany in 2004 (nucleotide percentage distance 4.7% for the SIDS specimen and 0.9% for the CSF specimen). In the structural protein gene fragment, the closest relative of both viruses was a strain obtained in the Netherlands in 2008 (Nijmegen2008, nucleotide distance 13.9% for the SIDS specimen and 3.5% for the CSF specimen). This suggested geographic rather than phylogenetic clustering of viruses detected within and beyond the respiratory and enteric tracts. However, formal and final virus typing is pending because VP1 regions could not be sequenced from 2 viruses.

**Figure:**
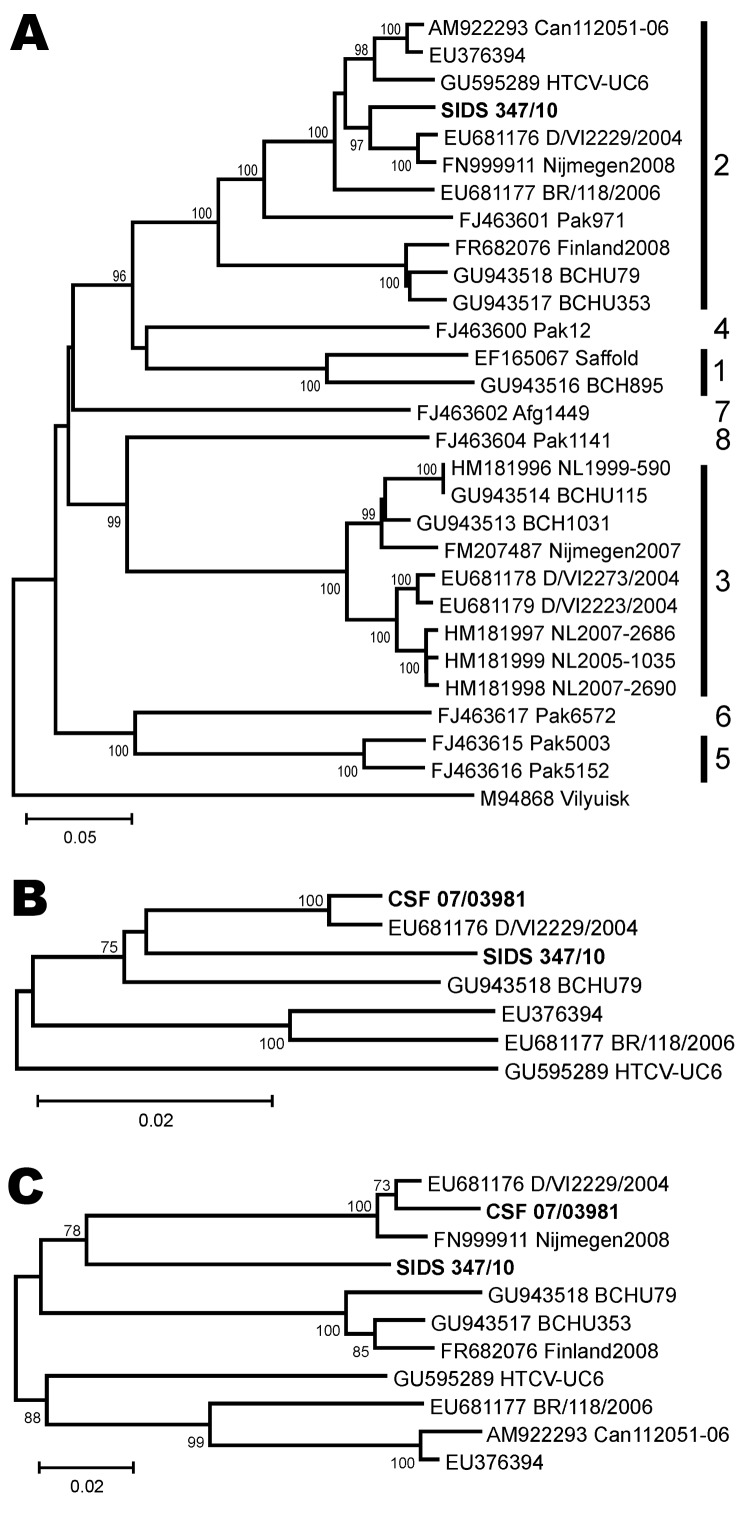
Human cardiovirus phylogeny including novel viruses from myocardial tissue and cerebrospinal fluid. A) The 798-nt complete viral protein (VP) 1 phylogeny, with genotypes indicated to the right. Vilyuisk virus was used as an outgroup. B) The 802-nt partial 5′ untranslated region phylogeny of genotype 2 human cardioviruses. C) The 489-nt complete leader, complete VP4 and partial VP2 phylogeny of genotype 2 human cardioviruses. Neighbor-joining phylogenies were calculated with MEGA5 (www.megasoftware.net) by using a percentage nucleotide distance substitution model with complete deletion of gaps and 1,000 bootstrap reiterations for confidence testing. Only bootstrap values >70% are shown at node points. Scale bars indicate percentage nucleotide distance. Novel viruses from this study (sudden infant death syndrome [SIDS] 347/10 and cerebrospinal fluid [CSF] 07/03981) are shown in **boldface**. Reference viruses are given with GenBank accession number and strain name (when available).

Absence of other detectable pathogens in 1 of the meningoencephalitis case-patients (07/03981) made causation by hCV plausible (Table A1). For the second case (VI1607), an enterovirus was co-detected by real-time RT-PCR in CSF and feces. Serotyping from feces classified this virus as echovirus type 30, known to cause aseptic meningitis. For the specimen from the child who died of SIDS, a rhinovirus was co-detected at low concentrations (real-time RT-PCR threshold cycle value >40), most compatible with shedding after previous respiratory infection (*9*).

## Conclusions

The detection of hCVs in body compartments beyond the respiratory and enteric tracts is novel and suggests a role of these viruses in organ-related disease. A low detection rate in CSF does not contradict a general potential of these viruses to cause meningoencephalitis, as exemplified by enteroviruses for which lack of detection in CSF despite clear association with disease is not uncommon (*10*). Considering links between the related *Theilovirus* and demyelinating disease in laboratory models (*1*), long-term outcomes of patients with hCV infection of the central nervous system should be followed up. Such longitudinal studies should include sufficient numbers of patients because natural infections with *Theilovirus* in rodents are common and will less frequently result in multiple sclerosis–like disease than in laboratory models (*1*). The rarity of hCV detection in our study suggests the assembly of such cohorts to be a difficult and lengthy task that could benefit greatly from international coordination.

Despite the absence of histopathologic alterations, the detection of hCV in a child who died of SIDS is remarkable because the related encephalomyocarditis virus constitutes a prototypic model for myocarditis in mammals (*11*). Again, the high human seroprevalence against hCV (*12*) will complicate epidemiologic studies, yet investigations of links between hCV and SIDS are highly justified because diarrhea is an acknowledged risk factor for SIDS (*13*).

A limitation of our study is that the VP1 genomic region of the viruses detected in CSF could not be obtained. In analogy to enteroviruses and parechoviruses, certain genotypes may be associated with distinct disease profiles, like polioviruses with encephalitis or parechovirus 3 with meningitis (*14*). Although we were able to classify the virus detected in the child who died of SIDS as a common genotype 2, the partial hCV sequence from a patient with meningitis did not permit typing because hCVs, as all picornaviruses, recombine frequently (*15*). We thus cannot exclude that the viruses detected in the meningitis cases may have acquired distinct features in their capsid protein or elsewhere that might influence pathogenicity.

## Appendix

**Table A1:** Characteristics of patients with positive human cardiovirus test results, Germany*

Sample ID	Sampling location/ date/cohort	Clinical diagnosis	Patient age/ sex	Leading symptoms	Body temperature, °C	Recent history	General medical history	Symptomatic contact person	Altered serum laboratory parameters	Lumbar puncture results	Clinical course	Virus concentration, RNA copies per mL/g specimen	Co-infections†	Main pathologic autopsy finding
07/03981	Bonn/Feb 2007/ virologic routine diagnostics	Meningitis	9 y/F	Severe frontal headache, fatigue, meningism, photosensitivity	38.2	Sore throat 1 wk earlier	Uneventful	None	C-reactive protein (2.1 mg/L)	Cells 582/µL (lymphocytes 368/µL, neutrophils 162/µL, monocytes 50/µL), protein 434 mg/L, glucose 58 mg/dL	Antibiosis (cefotaxime/ ceftazidime) for 14 d, improvement after 5 d, discharged healthy after 14 d	1.14 × 10^4^/mL CSF, 9.50 × 10^2^/g feces	None	NA
VI1607	Hamburg/ Jul 2007/ virologic routine diagnostics	Meningitis	3 y/F	Reduced general condition, meningism, emesis	38.1/39.6 (d 1)	Family vacation at German seaside	Uneventful	None	C-reactive protein (13 mg/L)	Cells 16/µL, protein 237 mg/L	Antibiosis (ceftriaxone) for 3 d, discharged healthy after 5 d	9.63 × 10^2^/mL CSF	Enterovirus ECHO30	NA
347/10	Bonn/2010/ forensic routine diagnostics	SIDS‡	9 mo/M	Rigor mortis, paleness	39.2 (rectal)	Obstructive bronchitis, gastroenteritis (rotavirus), skin infection (*Staphylococcus aureus*)	Preterm birth (wk 31 of pregnancy), birth weight 1,400 g, partial trisomy 6, partial monosomy 20, hypothyreosis, albinism, retrognathia, dystrophy	NA	NA	NA	NA	Below assay detection limit	Rhinovirus	Unclear cause of death, aspiration of gastric contents

*CSF, cerebrospinal fluid; NA, not applicable; SIDS, sudden infant death syndrome. †Fecal specimens were tested for norovirus and enteroviruses by real-time reverse transcription PCR (RT-PCR) and for rotavirus, adenovirus, and astroviruses by ELISA; CSF specimens were tested by real-time RT-PCR/PCR/nested RT-PCR for enterovirus, influenza virus, parechovirus, rhinovirus, tick-borne encephalitis virus, coronavirus, adenovirus, parvovirus, mumps, measles, and all other human paramyxoviruses and the human herpesviruses HSV1/2, varicella zoster virus, cytomegalovirus, Epstein-Barr virus, and human herpesvirus 6. Initial and follow-up serum specimens were tested for lymphocytic choriomeningitis virus by complement binding reaction. Diagnostics of bacterial pathogens included ELISA, Western blot, and PCR for *Borrelia burgdorferi* sensu lato and *Treponoma pallidum*, complement binding reaction for *Leptospira* spp., and standard microbiologic culture methods. ‡Because of partial trisomy 6 and monosomy 20, also classifiable as sudden unexpected death in infancy.
